# The Additional Prognostic Value of Serial Biomarker Measurements for Extubation Failure Among Patients With COVID-19 Acute Respiratory Distress Syndrome

**DOI:** 10.1177/11772719251385929

**Published:** 2025-11-12

**Authors:** Carline N. L. Groenland, Adinde H. Siemers, Eric A. Dubois, Diederik Gommers, Leo Heunks, Evert-Jan Wils, Vivan J. M. Baggen, Henrik Endeman

**Affiliations:** 1 Department of Intensive Care, Erasmus MC, Rotterdam, The Netherlands; 2 Department of Cardiology, Thorax Center, Cardiovascular Institute, Erasmus MC, Rotterdam, The Netherlands; 3 Department of Intensive Care, Radboud University Medical Center, Nijmegen, The Netherlands; 4 Department of Intensive Care, Franciscus Gasthuis & Vlietland Ziekenhuis, Rotterdam, The Netherlands; 5 Department of Intensive Care, OLVG, Amsterdam, The Netherlands

**Keywords:** extubation failure, mechanical ventilation, high-sensitivity troponin-T, N-terminal pro–B-type natriuretic peptide, interleukin-6, procalcitonin, biomarkers, repeated measurements, COVID-19

## Abstract

**Background:**

Extubation failure is associated with adverse outcomes in critically ill patients. While single biomarker measurements can aid prediction, repeated biomarker measurements can help to timely recognize underlying diseases.

**Objectives:**

The aim of this study was to investigate the temporal evolution of cardiac (N-terminal pro–B-type natriuretic peptide [NT-proBNP], high-sensitivity troponin T [Hs-TnT]) and inflammatory biomarkers (interleukin-6 [IL-6] and procalcitonin [PCT]) prior to extubation and determine their additional prognostic value.

**Design:**

Retrospective cohort study.

**Methods:**

Patients with COVID-19 extubated after mechanical ventilation were included. Daily biomarker levels were collected up to 3 days before extubation. The primary endpoint was extubation failure, defined as reintubation or death within 7 days. Linear mixed-effect models were used to analyze biomarker trajectories in patients with extubation success and failure. Each day before extubation a logistic regression model (consisting of the 4 biomarkers) was constructed to determine the model with the best discriminative ability.

**Results:**

Among 297 patients, 21.5% experienced extubation failure. Log_2_ Hs-TnT, NT-proBNP and PCT were higher on all days in patients with extubation failure (*P* < .001, *P* = .01, *P* = .01, respectively), whereas log_2_ IL-6 was not (*P* = .54). There was no difference in the change of biomarkers over the days between patients with extubation success and failure (*P*-value for interaction = .11, *P* = .82, *P* = .31, *P* = .84, respectively). The performance of the logistic regression model including the 4 biomarkers on the day of extubation was significantly better than the model 3 days before extubation (AUC 0.71, 95% CI: 0.64-0.79 vs AUC 0.66, 95% CI: 0.58-0.73, *P* = .03).

**Conclusion:**

Hs-TnT, NT-proBNP and PCT measured on the days before extubation are consistently higher in patients with extubation failure. However, there was no relation between the change in biomarker levels over time and extubation outcome. The serial assessment of Hs-TnT, NT-proBNP, PCT, and IL-6 do not seem to add prognostic information to predict extubation failure.

## Introduction

Predicting extubation failure remains challenging, as up to 20% of patients fail despite a successful spontaneous breathing trial (SBT).^1,2^ Delayed or unsuccessful weaning is associated with unfavorable outcomes, driving health care professionals to seek better predictors.^3-6^ Biomarkers are objective, usually rapidly available and quantifiable measures of underlying diseases which can be used to assess a patient’s readiness to wean. Previous studies showed the prognostic value of a single cardiac or inflammatory biomarker measurement such as Hs-TnT, (NT-pro)BNP, IL-6 and procalcitonin in predicting extubation failure.^7-11^

Repeatedly measured biomarkers may provide additional prognostic information, as was for instance shown for delta BNP measurements during the SBT and BNP driven fluid-management to wean from mechanical ventilation.^8,9,12-14^ Common causes of extubation failure are new (due to infection) or ongoing inflammation, fluid overload, and impaired cardiac function due to ischemia or congestive heart failure.^12,15-17^ These processes are dynamic and biomarkers reflecting these processes are subjected to biological variation. If abnormal biomarker trends can be identified in time, treatment can be initiated or anticipated, which may even limit the duration of weaning from mechanical ventilation and increase the chance of successful extubation. Therefore, we hypothesized that serial assessment of the trend of the biomarkers prior to extubation provides additional prognostic value to predict extubation failure.

The aim of this study was to investigate the temporal evolution of cardiac (NT-proBNP and Hs-TnT) and inflammatory (IL-6 and PCT) biomarkers prior to extubation and to determine the additional prognostic value of these serial measurements in patients with COVID-19 acute respiratory distress syndrome (C-ARDS).

## Methods

This retrospective cohort study was performed at the Erasmus MC, Rotterdam, the Netherlands. Patients were included between February 28th, 2020 and March 31st, 2022. Inclusion criteria were: Mechanically ventilated adult (⩾18 years) ICU patients, C-ARDS (defined according to the Berlin criteria^18^) as primary reason for ICU admission, extubation attempt (after successful completion of a 30-minute spontaneous breathing trial with a T-piece^19^). Exclusion criteria were: do not reintubate order, terminal illness (extubation as part of end-of-life care), transfer to another hospital before extubation attempt, tracheostomy before an extubation attempt, pregnant or post-partum women. Ethical approval of the study was obtained by the Institutional research Review Board of the Erasmus MC (MEC-2022-0740). This study was performed under exception from informed consent, as a waiver was provided for research including COVID-19 patients (MEC-2020-0381). Patients with registered objections were exempted from this waiver. This study is reported according to the STROBE statement Checklists—STROBE (strobe-statement.org) (Supplemental Table 1). Study details have been published previously.^7^

Extubation failure was defined according to the WIND definition: the need for reintubation or death within the next 7 days after extubation, regardless of whether post-extubation respiratory support was used or not.^20^ Patients who were not reintubated or death within 7 days were defined as extubation success.

During the COVID-19 pandemic, the routine morning laboratory measurements were extended with cardiac biomarkers (Hs-TnT, NT-proBNP) and inflammatory biomarkers (PCT, IL-6). We retrospectively collected the biomarkers that were measured on the 3 days preceding extubation and on the day of extubation. All biomarkers were measured on the Cobas 8000 analyzer (Roche Diagnostics, Mannheim, Germany). Patient characteristics were collected on the day of extubation. The following cut-off values were used to define elevated biomarker levels: Hs-TnT ⩾14 ng/L, NT-proBNP ⩾15 pmol/L, PCT ⩾0.25 ng/mL and IL-6 ⩾75 pg/mL.

### Statistical Analyses

Patient characteristics are presented as mean ± standard deviation or median [interquartile range, IQR], depending on the distribution of data. Categorical variables were described as frequency (percentage). Continuous variables between patients with extubation success and failure were compared using the Student’s *t*-test or Mann-Whitney *U* test, depending on the distribution of the data. Categorical variables were compared using the Pearson’s Chi-square test. Continuous variables among the different biomarker groups were compared using ANOVA or the Kruskal-Wallis test, depending on the distribution of the data. Categorical variables were compared using the Chi-square or Fisher exact test when applicable. Because of the highly skewed biomarker distributions, all biomarker values were log_2_ transformed for further analyses.

To investigate whether repeated biomarker measurements the days before extubation were associated with extubation failure, we constructed multiple models.

First, we constructed linear mixed effect models to describe the temporal evolution of biomarkers the days before extubation in relation to extubation success and failure. We included both a random intercept and a random slope per patient. The random intercept allows each patient to have a different baseline value of the biomarker. The random slope for days allows each patient to have their own rate of change in the biomarker over time. In the Supplemental Table 2 the constructed models are described in more detail. An interaction effect was included between days and extubation failure to assess whether the slope of the biomarker was different in patients with extubation failure compared to patients with extubation success.

Additionally, we determined whether the change in biomarker values the days before extubation was associated with extubation failure. To determine the change, we calculated the slope per patient per biomarker and investigated whether the slope was associated with extubation failure by calculating the odds ratio. The slope originated from the linear mixed model.

Secondly, to investigate on which day the combined panel of biomarkers has the best discriminative ability to predict extubation failure we constructed a logistic regression model for each day before extubation (4 logistic regression models in total). Each model consisted of the 4 biomarkers included in this study (Hs-TnT, NT-proBNP, PCT and IL-6).

The first logistic regression model included the biomarkers measured 3 days before extubation, the second model 2 days before extubation, the third model 1 day before extubation, and the fourth model on the day of extubation. For each model we calculated the area under the curve (AUC) with confidence intervals. The DeLong test was used to compare the AUC between the different logistic regression models.

Third, we constructed a logistic regression model to predict extubation failure for each biomarker separately. Patients were divided into 4 categories based on the last 2 biomarker values before extubation. According to the pre-defined clinical cut-off values, patients were divided into different groups; low-low, high-low, low-high and high-high.

Completeness of data on biomarkers is reported in Supplemental Table 3. Missing data was imputed using predictive mean matching^21^ using the mice package in R software, generating a single imputed dataset given the low proportion of missing values.

Two-sided *P* values of <.05 were considered statistically significant. Data analysis was performed using R software for statistical computing version 4.2.1.

## Results

### Patients’ Characteristics

Of the 599 screened patients, 297 patients met the inclusion criteria (Figure S1). The baseline characteristics are presented in Table 1. Both Figure S1 and Table 1 correspond to those in our previous paper.^7^ Patients with extubation failure were older, had a higher SOFA score on the day of extubation and a longer duration of invasive mechanical ventilation (IMV) before extubation. Extubation failure occurred in 64 patients (21.5%). NT-proBNP, Hs-TnT and PCT measured on the day of extubation were higher in patients with extubation failure. Additionally, in Supplemental Tables 4 to 7 baseline characteristics are presented based on the biomarker change in the last 2 days. Patients with a constant high Hs-TnT, NT-proBNP and PCT were (in comparison to the other groups) older, had more often hypertension, a longer duration of IMV, and higher SOFA scores on the day of extubation. These differences were not observed for IL-6. Smaller number of patients changed from a low biomarker level to a high biomarker level and vice versa.

**Table 1.** Baseline Characteristics.

Baseline characteristics	All patients (N = 297)	Extubation success (N = 233)	Extubation failure (N = 64)	*P*-value
Age (years)	60 [51-67]	59 [51-67]	64 [55-68]	.03
Male sex, N (%)	208 (70.0)	162 (69.5)	46 (71.9)	.85
BMI (kg/m^2^)	29.7 (±5.8)	29.9 (±5.7)	28.6 (±5.8)	.10
APACHE IV score	63 (±39)	62 (±43)	67 (±20)	.45
Charlson Comorbidity Index	2.0 [1.0-3.0]	2.0 [1.0-3.0]	3.0 [2.0-3.3]	.06
Comorbidities, N (%)				
Congestive heart failure	8 (2.7)	5 (2.1)	3 (4.7)	.50
Myocardial infarction	9 (3.0)	6 (2.6)	3 (4.7)	.64
PVD	10 (3.4)	8 (3.4)	2 (3.1)	1.00
Hypertension	117 (39.4)	86 (36.9)	31 (48.4)	.14
Chronic kidney disease	19 (6.4)	12 (5.2)	7 (10.9)	.17
Diabetes	81(27.2)	59 (25.3)	22 (34.3)	.33
Duration of IMV before extubation (days)	9 [6-13]	8 [6-11]	11 [7-15]	.001
SOFA score on the day of extubation	3 [2-5]	3 [2-4]	5 [3-7]	<.001
Total fluid balance until extubation (L)	0.9 [−1.0-3.0]	0.9 [−1.0-2.8]	1.0 [−0.3-3.3]	.35
Fluid balance 24-hours before extubation (L)	−0.4 [−1.0-0.2]	−0.4 [−1.1-0.2]	−0.3 [−0.9-0.2]	.63
Biomarker levels on the day of extubation				
Hs-TnT (ng/L)	14 [10-25]	13 [9-22]	20 [12-55]	<.001
NT-proBNP (pmol/L)	27 [12-62]	25 [12-57]	44 [16-91]	.01
Procalcitonin (ng/mL)	0.11 [0.06-0.22]	0.09 [0.06-0.17]	0.18 [0.10-0.39]	<.001
IL-6 (pg/mL)	74 [21-210]	78 [20-209]	68 [24-233]	.91

Abbreviations: APACHE IV, Acute Physiology And Chronic Health Evaluation IV; BMI, body mass index; Hs-TnT, high-sensitivity troponin T; IL-6, interleukin-6; IMV, invasive mechanical ventilation; NT-proBNP, N-terminal pro-B-type natriuretic peptide; PCT, procalcitonin; PVD, peripheral vascular disease; SOFA, Sequential Organ Failure Assessment.

Continuous variables are presented as mean ± standard deviation or median [interquartile range, IQR], as appropriate. Categorical variables are presented as N (%).

### Evolution of Biomarkers in the Days Preceding Extubation

**Figure 1.**
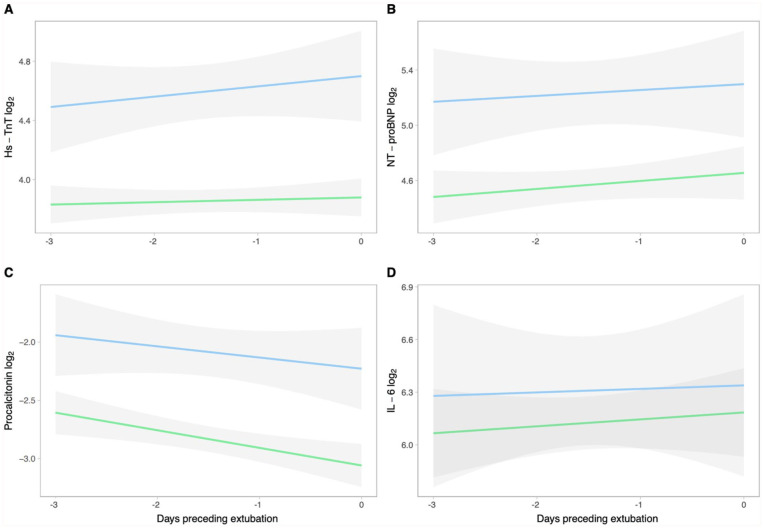
Evolution of biomarkers in the days preceding extubation, stratified by extubation outcome. Evolution of log_2_ Hs-TnT (A), log_2_ NT-proBNP (B), log_2_ PCT (C) and log_2_ IL-6 (D) in the days preceding extubation in patients with extubation success (green) and extubation failure (blue). *Y*-axis: the log_2_ value with its confidence interval; *X*-axis: the days preceding extubation.

The temporal evolution of biomarkers the days before extubation in relation to extubation outcome are depicted in Figure 1. The log_2_ Hs-TnT, NT-proBNP and PCT were consistently higher in patients with extubation failure (*P* < .001, *P* = .01, *P* = .01, respectively). The log_2_ IL-6 was not different in patients with success and failure (*P* = .54). There was no difference in the change of log_2_ Hs-TnT, NT-proBNP, PCT and IL-6 over time between patients with extubation success and failure (*P*-value for interaction = .11, *P* = .82, *P* = .31, *P* = .84, respectively). Additionally, to further assess whether the evolution of the biomarkers the days before extubation was associated with extubation failure, we calculated the slope based on the serial measurements. None of the slopes of the biomarkers were associated with extubation failure. In Supplemental Table 8 the ORs are shown.

### Predictive Performance of Biomarker Models on the Days Preceding Extubation

**Figure 2.**
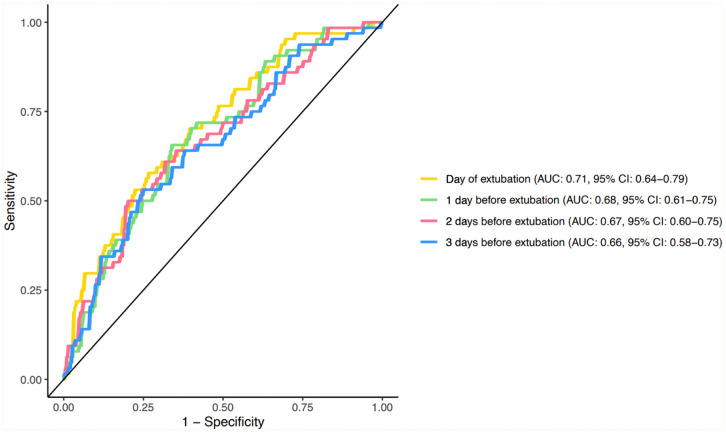
Predictive performance of biomarker models on the days preceding extubation. Receiver operating curve for each logistic regression model per day before and on the day of extubation. Abbreviation: AUC, area under the curve.

To investigate on which day the combination of biomarker levels had the best discriminative ability to predict extubation failure we constructed multiple logistic regression models for each day prior to and on the day of extubation. The AUCs ranged between 0.71 (95% CI: 0.64-0.79) (on the day of extubation) and 0.66 (95% CI: 0.58-0.73) (3 days before extubation), with overlapping confidence intervals (Figure 2). The performance of the model on the day of extubation was significantly better than the performance of the model 3 days before extubation (ΔAUC = 0.05, *P* = .03; Supplemental Table 9). There was no significant difference between the models when tested to their adjacent day.

### Prediction of Extubation Failure Using Clinical Cut-Off Values of Biomarkers

In Table 2 the odds ratios for extubation failure are presented for each biomarker separately. Patients with consistently high Hs-TnT, NT-proBNP and/or PCT levels had a significantly higher risk of extubation failure compared with the reference group (patients with consistently low levels). The ORs for patients with a decrease or increase in biomarker level were not significant.

**Table 2.** Odds Ratios for Extubation Failure Per Biomarker (Based On Clinical Cut-Off Values).

Change in biomarker category	N	ORs (95% CI)
Hs-TnT		
Reference group (Low → Low)	139	1.00
High → Low	13	0.44 (0.02-2.42)
Low → High	14	1.45 (0.30-5.10)
High → High	131	2.17 (1.21-3.97)
NT-proBNP		
Reference group (Low → Low)	152	1.00
High → Low	25	1.33 (0.41-3.68)
Low → High	22	2.00 (0.66-5.43)
High → High	98	2.24 (1.21-4.17)
Procalcitonin		
Reference group (Low → Low)	217	1.00
High → Low	10	1.22 (0.18-5.10)
Low → High	9	3.89 (0.93-15.40)
High → High	61	2.55 (1.34-4.81)
IL-6		
Reference group (Low → Low)	130	1.00
High → Low	20	1.16 (0.35-3.29)
Low → High	19	0.65 (0.14-2.13)
High → High	128	0.93 (0.51-1.68)

Patients were divided into groups based on the change of the biomarker. These groups were defined as follows: patients with a low biomarker value both the day before and on the day of extubation were classified as low-low (the reference group); patients with a high value the day before and a low value on the day of extubation as high-low; patients with a low value the day before and a high value on the day of extubation as low-high; and patients with a high value on both days as high-high. Cut-off values per biomarker were, respectively: Hs-TnT <14 ng/, NT-proBNP <15 pmol/L, PCT <0.25 ng/mL, IL-6 <4.4 pg/mL. The odds ratio (OR) was calculated against the reference group (low-low). N = number of patients per group.

## Discussion

In this study we assessed the association between serial cardiac and inflammatory biomarkers and extubation failure in patients with COVID-19 ARDS. We found that Hs-TnT, NT-proBNP and PCT on the days before extubation were consistently higher in patients with extubation failure. There was no significant increase or decrease in biomarkers the days before extubation in patients with extubation failure compared with extubation success. The predictive performance of biomarker models was highest on the day of extubation, with a significantly better discriminative ability compared to 3 days prior. We could not demonstrate additional prognostic value on top of a single measurement.

Although the discriminative ability of biomarkers to predict extubation failure was highest on the day of extubation, the AUC of the model 1 or 2 days before extubation was not significantly different and does thus provide some prognostic information. Although outside the scope of this study, measuring the biomarkers earlier before extubation could potentially alert clinicians to alter treatment and minimize the risk of extubation failure. Recent literature has elaborated on how different tracts can play a role in failure and how to discover and treat underlying illnesses causing failure.^10,22^ Early identification of a new or ongoing infection or inflammation may alert pro-actively reconsideration of antibiotics and/or anti-inflammatory agents like corticosteroids. Identification of signs of fluid overload or cardiac ischemia draws attention to intensification of diuretics,^8^ initiation of antihypertensive medication or anti-ischemic therapy. Such an approach may ultimately contribute to accelerated weaning. Timely measuring biomarkers and subsequent altering treatment aligns well with recent advances to perform aggressive screening for performing the SBT to limit the duration of weaning from mechanical ventilation.^23^

### Serial Cardiac Biomarkers

In our study serial Hs-TnT and NT-proBNP were consistently higher on the days before extubation in patients with extubation failure, and there was no significant increase or decrease. In the MaastriCCht cohort, the value of serial biomarker measurements during ICU admission in patients with COVID-19 was investigated, but in relation to survival.^24^ Log NT-proBNP decreased significantly in survivors versus non-survivors, while log Hs-TnT did not change over time. In our study we did not show a decrease in patients with extubation success. However, apart from the different study endpoint, also the timeframe of measurements was shorter (4 days) in comparison to the MaastriCCht cohort (whole ICU admission, median of 4 weeks), possibly explaining differences. In another study, when examining the change in BNP over a much shorter time interval (specifically during the SBT) in relation to extubation failure,^14^ the value of a relative change of BNP was demonstrated. In this case, the change in BNP can be attributed to the effort exerted during an SBT.

### Serial Inflammatory Biomarkers

In our study, where PCT was measured on the days before and on the day of extubation, no significant change over time was observed. However, PCT was consistently higher in patients with extubation failure. This is in line with previous literature showing that PCT is associated with the duration of mechanical ventilation and failure of liberation from oxygen therapy at day 28 after admission.^25,26^ Moreover, the clinical significance of the change rate of PCT has been investigated in patients with acute respiratory failure due to an acute exacerbation of COPD.^27^ In this Randomized Controlled Trial, patients in the intervention group were extubated based on more than a 50% change in PCT level (from admission). This approach yielded in a significant reduction of duration of invasive mechanical ventilation in comparison to the control group with conventional weaning strategies (2.10 ± 1.02 days vs 4.53 ± 1.28 days), without increasing the reintubation rate. As hypothesized in an earlier study of Alladina et al,^9^ inflammatory biomarkers might be of additional value in the assessment whether patients are ready to wean, since the assessment solely based on ventilator settings might not accurately represent the heterogenous damage in the lungs. Lower levels of IL-6 and ST-2 (measured at the early phase of ARDS) were associated with a shorter duration of mechanical ventilation. However, the authors did not investigate the change in these biomarkers over time in relation to the outcome. Future studies could examine the value of the relative change in biomarker levels between ICU admission and extubation, as such dynamics may capture ongoing pathological processes that could negatively affect weaning outcomes. For example, ongoing sepsis represented by increased inflammatory markers such as IL-18 and IL-18BP were found to be associated with SBT failure.^28^ This highlights the relevance of inflammatory markers in indicating ongoing inflammation, which may delay successful weaning from mechanical ventilation.

### Strengths and Limitations

This is the first study that repeatedly measured cardiac and inflammatory biomarkers the days before extubation and is therefore a contribution to our previously published paper.^7^ We used complementary statistical methods to determine the temporal evolution and prognostic value of repeated measurements. The percentage of missing data of biomarkers was low and multiple imputation was performed using predictive mean matching.

Our study has several limitations. Since the retrospective nature of the study, the moment of extubation was already known, which also allowed us to define the days before extubation. However, the timing of extubation depends, among other factors, on meeting ready-to-wean criteria and passing an SBT and is not planned days in advance. This complicates the determination of the optimal timing for biomarker measurements in clinical practice. Nevertheless, since there is no difference in the discriminative ability of the biomarkers measured on the day of extubation, and 1 or 2 days before extubation, there is a wide time range of when the biomarkers can be measured. Second, physicians were not blinded to the biomarker values so we cannot formally exclude that abnormal biomarker values may have influenced extubation decision-making. However, at that time, the value and clinical relevance of biomarker levels in relation to extubation were not well established and no clinical guidance on handling biomarker values was available or implemented. Third, the sample size of the patients with a change in biomarker from high-low and low-high is limited, which makes it challenging to draw firm conclusions from these results. Finally, as differences in biomarkers between classical ARDS and C-ARDS have been reported,^29^ future studies need to confirm our results in non-C-ARDS patients.

## Conclusion

This study shows that cardiac and inflammatory biomarkers measured on the days before extubation (Hs-TnT, NT-proBNP and PCT) are consistently higher in patients with extubation failure compared with extubation success. There was, however, no significant change in biomarker levels over time in relation to extubation failure, and only few patients seem to have clinically relevant fluctuations in biomarker levels. When analyzed using various methods, the serial assessment of Hs-TnT, NT-proBNP, PCT and IL-6 in the days before extubation does therefore not seem to add prognostic information to predict extubation failure. Nevertheless, an earlier measurement of Hs-TnT, NT-proBNP and PCT might facilitate more aggressive weaning strategies and could help enable timely treatment adjustments to shorten the duration of mechanical ventilation.

## Supplemental Material

sj-docx-1-bmi-10.1177_11772719251385929 – Supplemental material for The Additional Prognostic Value of Serial Biomarker Measurements for Extubation Failure Among Patients With COVID-19 Acute Respiratory Distress Syndrome Supplemental material, sj-docx-1-bmi-10.1177_11772719251385929 for The Additional Prognostic Value of Serial Biomarker Measurements for Extubation Failure Among Patients With COVID-19 Acute Respiratory Distress Syndrome by Carline N. L. Groenland, Adinde H. Siemers, Eric A. Dubois, Diederik Gommers, Leo Heunks, Evert-Jan Wils, Vivan J. M. Baggen and Henrik Endeman in Biomarker Insights
