# Supplementary material for: The Additional Prognostic Value of Serial Biomarker Measurements for Extubation Failure Among Patients With COVID-19 Acute Respiratory Distress Syndrome
Source: Biomark Insights. 2025 Nov 12;20:11772719251385929. doi: 10.1177/11772719251385929 (PMC12612537; doi:10.1177/11772719251385929)
Supplement: sj-docx-1-bmi-10.1177_11772719251385929 – Supplemental material for The Additional Prognostic Value of Serial Biomarker Measurements for Extubation Failure Among Patients With COVID-19 Acute Respiratory Distress Syndrome [file sj-docx-1-bmi-10.1177_11772719251385929.docx]

**Table S1 - STROBE Statement** —Checklist of items that should be included in reports of *cohort studies*

|  | Item No | Recommendation | Page No |
| --- | --- | --- | --- |
| **Title and abstract** | 1 | (*a*) Indicate the study’s design with a commonly used term in the title or the abstract | 1-3 |
|  |  | (*b*) Provide in the abstract an informative and balanced summary of what was done and what was found |  |
| Introduction | | | |
| Background/rationale | 2 | Explain the scientific background and rationale for the investigation being reported | 4 |
| Objectives | 3 | State specific objectives, including any prespecified hypotheses | 4 |
| Methods | | | |
| Study design | 4 | Present key elements of study design early in the paper | 5 |
| Setting | 5 | Describe the setting, locations, and relevant dates, including periods of recruitment, exposure, follow-up, and data collection | 5 |
| Participants | 6 | (*a*) Give the eligibility criteria, and the sources and methods of selection of participants. Describe methods of follow-up | 5 |
|  |  | (*b*) For matched studies, give matching criteria and number of exposed and unexposed |  |
| Variables | 7 | Clearly define all outcomes, exposures, predictors, potential confounders, and effect modifiers. Give diagnostic criteria, if applicable | 5/6 |
| Data sources/ measurement | 8* | For each variable of interest, give sources of data and details of methods of assessment (measurement). Describe comparability of assessment methods if there is more than one group | 5/6 |
| Bias | 9 | Describe any efforts to address potential sources of bias | 7 |
| Study size | 10 | Explain how the study size was arrived at | 5 |
| Quantitative variables | 11 | Explain how quantitative variables were handled in the analyses. If applicable, describe which groupings were chosen and why | - |
| Statistical methods | 12 | (*a*) Describe all statistical methods, including those used to control for confounding | 6/7 |
|  |  | (*b*) Describe any methods used to examine subgroups and interactions |  |
|  |  | (*c*) Explain how missing data were addressed |  |
|  |  | (*d*) If applicable, explain how loss to follow-up was addressed |  |
|  |  | (*e*) Describe any sensitivity analyses |  |
| Results | | |  |
| Participants | 13* | (a) Report numbers of individuals at each stage of study—eg numbers potentially eligible, examined for eligibility, confirmed eligible, included in the study, completing follow-up, and analysed | 8 |
|  |  | (b) Give reasons for non-participation at each stage |  |
|  |  | (c) Consider use of a flow diagram |  |
| Descriptive data | 14* | (a) Give characteristics of study participants (eg demographic, clinical, social) and information on exposures and potential confounders | 8 |
|  |  | (b) Indicate number of participants with missing data for each variable of interest |  |
|  |  | (c) Summarise follow-up time (eg, average and total amount) |  |
| Outcome data | 15* | Report numbers of outcome events or summary measures over time | 8 |

| Main results | 16 | (*a*) Give unadjusted estimates and, if applicable, confounder-adjusted estimates and their precision (eg, 95% confidence interval). Make clear which confounders were adjusted for and why they were included | 8-9 |
| --- | --- | --- | --- |
|  |  | (*b*) Report category boundaries when continuous variables were categorized |  |
|  |  | (*c*) If relevant, consider translating estimates of relative risk into absolute risk for a meaningful time period |  |
| Other analyses | 17 | Report other analyses done—eg analyses of subgroups and interactions, and sensitivity analyses | - |
| Discussion | | | |
| Key results | 18 | Summarise key results with reference to study objectives | 10 |
| Limitations | 19 | Discuss limitations of the study, taking into account sources of potential bias or imprecision. Discuss both direction and magnitude of any potential bias | 12 |
| Interpretation | 20 | Give a cautious overall interpretation of results considering objectives, limitations, multiplicity of analyses, results from similar studies, and other relevant evidence | 10-13 |
| Generalisability | 21 | Discuss the generalisability (external validity) of the study results | 12 |
| Other information | | | |
| Funding | 22 | Give the source of funding and the role of the funders for the present study and, if applicable, for the original study on which the present article is based | 14 |

*Give information separately for exposed and unexposed groups.

**Note:** An Explanation and Elaboration article discusses each checklist item and gives methodological background and published examples of transparent reporting. The STROBE checklist is best used in conjunction with this article (freely available on the Web sites of PLoS Medicine a http://www.plosmedicine.org/, Annals of Internal Medicine at http://www.annals.org/, and Epidemiology at http://www.epidem.com/). Information on the STROBE Initiative is available at http://www.strobe-statement.org.

**Table S2: Linear mixed model per biomarker**

| **LMM per biomarker** |
| --- |
| lme(HsTnT_log2 ~ (Days*Reintubation), data = data_biocov, random = ~ Days\| id, method = "ML",  control = lmeControl(opt = "optim", maxIter = 1000)) |
| lme(NTproBNP_log2 ~ (Days*Reintubation), data = data_biocov, random = ~ Days\| id, method = "ML",  control = lmeControl(opt = "optim", maxIter = 1000)) |
| lme(PCT_log2 ~ (Days*Reintubation), data = data_biocov, random = ~ Days\| id, method = "ML",  control = lmeControl(opt = "optim", maxIter = 1000)) |
| lme(IL6_log2 ~ (Days*Reintubation), data = data_biocov, random = ~ Days\| id, method = "ML",  control = lmeControl(opt = "optim", maxIter = 1000)) |

**Table S3. Number of patients with missing biomarker values per day before extubation**

| Biomarkers | 3 days before extubation  N (%) | 2 days before extubation  N (%) | 1 day before extubation  N (%) | The day of extubation  N (%) |
| --- | --- | --- | --- | --- |
| Hs-TnT | 24 (8.1) | 18 (6.1) | 13 (4.4) | 9 (3.0) |
| NT-proBNP | 26 (8.8) | 18 (6.1) | 16 (5.4) | 11 (3.7) |
| Procalcitonin | 27 (9.1) | 17 (5.7) | 16 (5.4) | 10 (3.4) |
| IL-6 | 29 (9.8) | 19 (6.4) | 17 (5.7) | 10 (3.4) |

**Table S4. Baseline characteristics Hs-TnT, stratified by Hs-TnT course**

|  | Hs-TnT low 🡪 low (n=139) | Hs-TnT high 🡪 low (n=13) | Hs-TnT low 🡪 high (n=14) | Hs-TnT high 🡪 high (n=131) | P-value |
| --- | --- | --- | --- | --- | --- |
| Age (years) | 57 [47 – 65] | 61 [59 – 67] | 60 [47 – 65] | 65 [54 – 70] | 0.001 |
| Male sex, N (%) | 92 (66.2) | 12 (92.3) | 11 (78.6) | 93 (71.0) | 0.20 |
| BMI (kg/m2) | 29.5 (5.7) | 28.8 (3.2) | 30.4 (6.3) | 29.8 (6.0) | 0.89 |
| APACHE IV score | 56 [46 – 65] | 49 [48 – 65] | 50 [44 – 63] | 65 [57 – 76] | <0.001 |
| Charlson Comorbidity Index | 2.0 [0.5 – 3.0] | 3.0 [1.0 – 5.0] | 2.0 [0.0 – 3.0] | 3.0 [2.0 – 4.0] | <0.001 |
| Comorbidities |  |  |  |  |  |
| - Congestive heart failure | 3 (2.2) | 0 (0.0) | 0 (0.0) | 5 (3.8) | 0.68 |
| - Myocardial Infarction | 5 (3.6) | 1 (7.7) | 0 (0.0) | 3 (2.3) | 0.62 |
| - Hypertension | 44 (31.7) | 2 (15.4) | 1 (7.1) | 70 (53.4) | <0.001 |
| - PVD | 1 (0.7) | 0 (0.0) | 1 (7.1) | 8 (6.1) | 0.07 |
| - Chronic kidney disease | 3 (2.2) | 1 (7.7) | 0 (0.0) | 15 (11.5) | 0.01 |
| - Diabetes | 32 (23.0) | 3 (23.1) | 3 (21.4) | 43 (32.8) | 0.57 |
| Duration of IMV before extubation (days) | 8 [6 – 11] | 8 [7 – 13] | 8 [6 – 10] | 10 [7 – 14] | <0.001 |
| SOFA score on the day of extubation | 3 [2 – 4] | 3 [2 – 4] | 4 [2 – 5] | 4 [3 – 6] | <0.001 |
| Total fluid balance until extubation (L) | 1.0 [-0.6 – 3.2] | 1.1 [-1.6 – 3.1] | 0.4 [-1.8 – 2.4] | 0.9 [-1.1 – 2.5] | 0.60 |
| Fluid balance 24-hours before extubation (L) | -0.3 [-1.0 – -0.2] | -0.9 [-1.8 – 0.2] | -0.6 [-1,8— -0.4] | -0.4 [-0.9 – 0.1] | 0.14 |
| Extubation failure (%) | 22 (15.8) | 1 (7.7) | 3 (21.4) | 38 (29.0) | 0.04 |
| Death ICU (%) | 4 (2.9) | 0 (0.0) | 1 (7.1) | 4 (3.1) | 0.75 |
| Death hospital (%) | 4 (2.9) | 0 (0.0) | 2 (14.3) | 6 (4.6) | 0.18 |

Patients were divided into four groups based on the change in Hs-TnT. These groups were defined as follows: patients with a low biomarker value both the day before and on the day of extubation were classified as low-low (the reference group); patients with a high value the day before and a low value on the day of extubation as high-low; patients with a low value the day before and a high value on the day of extubation as low-high; and patients with a high value on both days as high-high. Hs-TnT low: <14 ng/L, Hs-TnT high: ≥14 ng/L. N= number of patients per group. Continuous variables are presented as mean ± standard deviation or median [interquartile range, IQR], as appropriate. Categorical variables are presented as N (%). Continuous variables were compared using ANOVA or the Kruskal-Wallis test and categorical variables were compared using the Chi-square or Fisher exact test. Abbreviations: BMI, Body Mass Index , APACHE IV, Acute Physiology And Chronic Health Evaluation IV, PVD, peripheral vascular disease, IMV, invasive Mechanical Ventilation, SOFA, Sequential Organ Failure Assessment, ICU, Intensive Care Unit

**Table S5. Baseline characteristics NT-proBNP, stratified by NT-proBNP course**

|  | NT-proBNP low 🡪 low (n=152) | NT-proBNP high 🡪 low (n=25) | NT-proBNP low 🡪 high (n=22) | NT-proBNP high 🡪 high (n=98) | P-value |
| --- | --- | --- | --- | --- | --- |
| Age (years) | 56 [46 – 65] | 59 [53 – 68] | 64 [58 – 70] | 65 [57 – 71] | <0.001 |
| Male sex, N (%) | 107 (70.4) | 18 (72.0) | 16 (72.7) | 67 (68.4) | 0.967 |
| BMI (kg/m2) | 29.7 (4.8) | 30.8 (9.4) | 28.7 (5.9) | 29.4 (5.9) | 0.59 |
| APACHE IV score | 55 [46 – 64] | 58 [52 – 63] | 64 [52 – 72] | 67 [58 – 76] | <0.001 |
| Charlson Comorbidity Index | 2.0 [0.5 – 3.0] | 3.0 [1.0 – 5.0] | 2.0 [0.0 – 3.0] | 3.0 [2.0 – 4.0] | <0.001 |
| Comorbidities |  |  |  |  |  |
| - Congestive heart failure | 2 (1.3) | 1 (4.0) | 0 (0.0) | 5 (5.1) | 0.257 |
| - Myocardial Infarction | 5 (3.3) | 0 (0.0) | 2 (9.1) | 2 (2.0) | 0.273 |
| - Hypertension | 49 (32.2) | 11 (44.0) | 5 (22.7) | 52 (53.1) | 0.003 |
| - PVD | 4 (2.6) | 0 (0.0) | 1 (4.5) | 5 (5.1) | 0.547 |
| - Chronic kidney disease | 3 (2.0) | 2 (8.0) | 0 (0.0) | 14 (14.3) | 0.001 |
| - Diabetes | 27 (17.8) | 10 (40.0) | 7 (31.8) | 37 (37.8) | 0.57 |
| Duration of IMV before extubation (days) | 8 [6 – 12] | 9 [7 – 13] | 8 [6 – 10] | 10 [7 – 14] | 0.015 |
| SOFA score on the day of extubation | 3 [2 – 4] | 3 [2 – 5] | 3.5 [2.25 – 5] | 4 [3 – 6] | 0.003 |
| Total fluid balance until extubation (L) | 0.9 [-1.08 – 3.08] | 0.0 [-0.85 – 2.07] | 1.7 [0.43 – 3.10] | 1.11 [-0.95 – 2.97] | 0.384 |
| Fluid balance 24-hours before extubation (L) | -0.41 [-1.00 – 0.19] | -0.095 [-0.817 – 151] | -0.56 [-1.32 – 0.08] | -0.46 [-1.03 – 0.98] | 0.760 |
| Extubation failure (%) | 24 (15.8) | 5 (20.0) | 6 (27.3) | 29 (29.6) | 0.066 |
| Death ICU (%) | 3 (2.0) | 2 (8.0) | 1 (4.5) | 3 (3.1) | 0.415 |
| Death hospital (%) | 3 (2.0) | 2 (8.0) | 1 (4.5) | 6 (6.1) | 0.284 |

Patients were divided into four groups based on the change in NT-proBNP. These groups were defined as follows: patients with a low biomarker value both the day before and on the day of extubation were classified as low-low (the reference group); patients with a high value the day before and a low value on the day of extubation as high-low; patients with a low value the day before and a high value on the day of extubation as low-high; and patients with a high value on both days as high-high. NT-proBNP low: <15 pmol/L, NT-proBNP high: ≥15 pmol/L. N= number of patients per group. Continuous variables are presented as mean ± standard deviation or median [interquartile range, IQR], as appropriate. Categorical variables are presented as N (%). Continuous variables were compared using ANOVA or the Kruskal-Wallis test and categorical variables were compared using the Chi-square or Fisher exact test. Abbreviations: BMI, Body Mass Index , APACHE IV, Acute Physiology And Chronic Health Evaluation IV, PVD, peripheral vascular disease, IMV, invasive Mechanical Ventilation, SOFA, Sequential Organ Failure Assessment, ICU, Intensive Care Unit

**Table S6. Baseline characteristics PCT, stratified by PCT course**

|  | PCT low 🡪 low (n=217) | PCT high 🡪 low (n=10) | PCT low 🡪 high (n=9) | PCT high 🡪 high (n=61) | P-value |
| --- | --- | --- | --- | --- | --- |
| Age (years) | 58 [48 – 66] | 64 [58 – 67] | 65 [61 – 66] | 65 [54 – 72] | 0.006 |
| Male sex, N (%) | 161 (74.2) | 6 (60.0) | 6 (66.7) | 35 (57.4) | 0.073 |
| BMI (kg/m2) | 29.8 (6.0) | 29.8 (4.6) | 31.5 (3.6) | 28.7 (5.4) | 0.43 |
| APACHE IV score | 58 [48 – 67] | 66 [55 – 68] | 64 [64 – 65] | 66 [53 – 86] | 0.008 |
| Charlson Comorbidity Index | 2.00 [1.00, 3.00] | 3.00 [2.25, 3.00] | 3.00 [3.00, 3.00] | 3.00 [2.00, 4.00] | <0.001 |
| Comorbidities |  |  |  |  |  |
| - Congestive heart failure | 5 (2.3) | 0 (0.0) | 0 (0.0) | 3 (4.9) | 0.614 |
| - Myocardial Infarction | 6 (2.8) | 2 (20.0) | 0 (0.0) | 1 (1.6) | 0.015 |
| - Hypertension | 78 (35.9) | 4 (40.0) | 3 (33.3) | 32 (52.5) | 0.134 |
| - PVD | 6 (2.8) | 0 (0.0) | 1 (11.1) | 3 (4.9) | 0.440 |
| - Chronic kidney disease | 9 (4.1) | 1 (10.0) | 0 (0.0) | 9 (14.8) | 0.021 |
| - Diabetes | 42 (19.4) | 5 (50.0) | 4 (44.4) | 30 (49.2) | <0.001 |
| Duration of IMV before extubation (days) | 8 [6 – 12] | 7 [7 – 11] | 13 [10 – 17] | 10 [7 – 15] | 0.025 |
| SOFA score on the day of extubation | 3 [2 – 4] | 3 [2 – 5] | 4 [3 – 5] | 5 [3 – 8] | <0.001 |
| Total fluid balance until extubation (L) | 0.93 [-0.99 – 2.97] | -0.17 [-2.84 – 2.16] | 1.60 [-2,10 – 3.67] | 1.10 [-0.51 – 3.08] | 0.393 |
| Fluid balance 24-hours before extubation (L) | -0.49 [-1.09 – 0.15] | -0.96 [-1.21— -0.78] | 0.05 [-0.31 – 0.19] | -0.23 [-0.72 – 0.37] | 0.022 |
| Extubation failure (%) | 37 (17.1) | 2 (20.0) | 4 (44.4) | 21 (34.4) | 0.010 |
| Death ICU (%) | 6 (2.8) | 0 (0.0) | 0 (0.0) | 3 (4.9) | 0.709 |
| Death hospital (%) | 7 (3.2) | 0 (0.0) | 0 (0.0) | 5 (8.2) | 0.274 |

Patients were divided into four groups based on the change in PCT. These groups were defined as follows: patients with a low biomarker value both the day before and on the day of extubation were classified as low-low (the reference group); patients with a high value the day before and a low value on the day of extubation as high-low; patients with a low value the day before and a high value on the day of extubation as low-high; and patients with a high value on both days as high-high. PCT low: <0.25 ng/mL, PCT high: ≥0.25 ng/mL. N= number of patients per group. Continuous variables

are presented as mean ± standard deviation or median [interquartile range, IQR], as appropriate. Categorical variables are presented as N (%). Continuous variables were compared using ANOVA or the Kruskal-Wallis test and categorical variables were compared using the Chi-square or Fisher exact test. Abbreviations: BMI, Body Mass Index , APACHE IV, Acute Physiology And Chronic Health Evaluation IV, PVD, peripheral vascular disease, IMV, invasive Mechanical Ventilation, SOFA, Sequential Organ Failure Assessment, ICU, Intensive Care Unit

**Table S7. Baseline characteristics IL-6, stratified by IL-6 course**

|  | IL-6 low 🡪 low (n=148) | IL-6 high 🡪 low (n=18) | IL-6 low 🡪 high (n=23) | IL-6 high 🡪 high (n=108) | P-value |
| --- | --- | --- | --- | --- | --- |
| Age (median [IQR]) | 61 [52 – 69] | 60 [52 – 65] | 50 [41 – 61] | 61 [53 – 66] | 0.006 |
| Male sex, N (%) | 100 (67.6) | 11 (61.1) | 19 (82.6) | 78 (72.2) | 0.073 |
| BMI (median [IQR]) | 29.4 (5.6) | 31.4 (6.5) | 29.9 (6.1) | 29.6 (5.7) | 0.59 |
| APACHEIV (median [IQR]) | 59 [48 – 68] | 57 [53 – 67] | 51 [46 – 63] | 62 [51 – 71] | 0.008 |
| Charlson Comorbidity Index | 2.0 [1.0 – 3.0] | 2.5 [1.3 – 4.0] | 1.0 [0.0 – 2.5] | 2.0 [1.0 – 3.0] | 0.049 |
| Comorbidities |  |  |  |  |  |
| - Congestive heart failure | 3 (2.0) | 1 (5.6) | 1 (4.3) | 3 (2.8) | 0.788 |
| - Myocardial Infarction | 8 (5.4) | 0 (0.0) | 0 (0.0) | 1 (0.9) | 0.124 |
| - Hypertension | 62 (41.9) | 9 (50.0) | 5 (21.7) | 41 (38.0) | 0.228 |
| - PVD | 3 (2.0) | 2 (11.1) | 0 (0.0) | 5 (4.6) | 0.141 |
| - Chronic kidney disease | 5 (3.4) | 3 (16.7) | 2 (8.7) | 9 (8.3) | 0.098 |
| - Diabetes | 42 (17.8) | 6 (40.0) | 4 (31.8) | 29 (37.8) | 0.78 |
| Duration of IMV before extubation (days) | 10 [6 – 14] | 10 [8 – 16] | 7 [6 – 10] | 8 [7 – 11] | 0.025 |
| SOFA score on the day of extubation | 3 [2 – 5] | 3.5 [2 – 5] | 3 [3 – 3.5] | 3 [2 – 5] | 0.703 |
| Total fluid balance until extubation (L) | 0.88 [-0.79 – 3.27] | 1.32 [-0.56 – 3.33] | 0.93 [-0.20 – 1.99] | 1.10 [-1.21 – 2.80] | 0.728 |
| Fluid balance 24-hours before extubation (L) | -0.31 [-0.95 – 0.31] | -0.02 [-0.51 – 0.17] | -0.59 [-1.20 – -0.30] | -0.55 [-1.08 – 0.03] | 0.158 |
| Extubation failure (%) | 35 (23.6) | 5 (27.8) | 2 (8.7) | 22 (20.4) | 0.371 |
| Death ICU (%) | 6 (4.1) | 1 (5.6) | 1 (4.3) | 1 (0.9) | 0.443 |
| Death hospital (%) | 7 (4.7) | 2 (11.1) | 1 (4.3) | 2 (1.9) | 0.279 |

Patients were divided into four groups based on the change in IL-6. These groups were defined as follows: patients with a low biomarker value both the day before and on the day of extubation were classified as low-low (the reference group); patients with a high value the day before and a low value on the day of extubation as high-low; patients with a low value the day before and a high value on the day of extubation as low-high; and patients with a high value on both days as high-high. IL-6 low: <75 pg/mL, IL-6 high: ≥75 pg/mL. N= number of patients per group. Continuous variables are presented as mean ± standard deviation or median [interquartile range, IQR], as appropriate. Categorical variables are presented as N (%). Continuous variables were compared using ANOVA or the Kruskal-Wallis test and categorical variables were compared using the Chi-square or Fisher exact test. Abbreviations: BMI, Body Mass Index , APACHE IV, Acute Physiology And Chronic Health Evaluation IV, PVD, peripheral vascular disease, IMV, invasive Mechanical Ventilation, SOFA, Sequential Organ Failure Assessment, ICU, Intensive Care Unit

**Table S8. ORs of slopes of the biomarkers**

|  | **Odds Ratio** | **P-value** |
| --- | --- | --- |
| Slope Hs-TnT (ng/L) | 1.003 (0.98 – 1.030) | 0.823 |
| Slope NT-proBNP (pmol/l) | 1.002 (0.998 – 1.012) | 0.253 |
| Slope Procalcitonin (ng/mL) | 1.038 (0.88 - 1.608) | 0.742 |
| Slope IL-6 (pg/mL) | 1.000 (0.997 – 1.001) | 0.801 |

**Table S9. Model performance per day before extubation**

|  | **AUC (95% CI)** | **Model 1**  **(p-value)** | **Model 2**  **(p-value)** | **Model 3**  **(p-value)** |
| --- | --- | --- | --- | --- |
| AUC model 1  (3 days before extubation) | 0.66 (0.58 – 0.73) | - | - | - |
| AUC model 2  (2 days before extubation) | 0.67 (0.60 – 0.75) | 0.45 | - | - |
| AUC model 3  (1 day before extubation) | 0.68 (0.61 – 0.75) | 0.24 | 0.58 | - |
| AUC model 4  (day of extubation) | 0.71 (0.64 – 0.79) | **0.03** | 0.10 | 0.07 |

The p-values presented are calculated based on the DeLong test between the differing models.


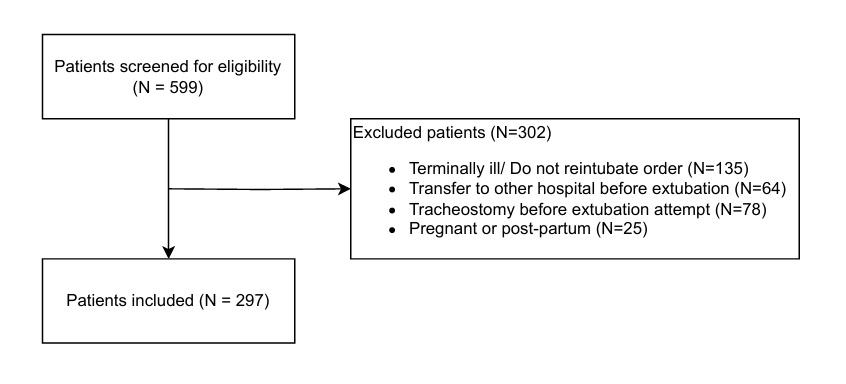


**Figure S1. Flowchart of patient selection**
